# High Diversity of Rabies Viruses Associated with Insectivorous Bats in Argentina: Presence of Several Independent Enzootics

**DOI:** 10.1371/journal.pntd.0001635

**Published:** 2012-05-08

**Authors:** Carolina Piñero, Federico Gury Dohmen, Fernando Beltran, Leila Martinez, Laura Novaro, Susana Russo, Gustavo Palacios, Daniel M. Cisterna

**Affiliations:** 1 Servicio de Neurovirosis, Instituto Nacional de Enfermedades Infecciosas, Administración Nacional de Laboratorios e Institutos de Salud (ANLIS) “Dr. Carlos G. Malbran,” Buenos Aires, Argentina; 2 Instituto de Zoonosis “Dr. Luis Pasteur,” Buenos Aires, Argentina; 3 Dirección de Laboratorio y Control Técnico, Servicio Nacional de Sanidad y Calidad Agroalimentaria, Buenos Aires, Argentina; 4 National Center for Biodefense and Infectious Diseases, George Mason University, Manassas, Virginia, United States of America; University of Texas Medical Branch, United States of America

## Abstract

**Background:**

Rabies is a fatal infection of the central nervous system primarily transmitted by rabid animal bites. Rabies virus (RABV) circulates through two different epidemiological cycles: terrestrial and aerial, where dogs, foxes or skunks and bats, respectively, act as the most relevant reservoirs and/or vectors. It is widely accepted that insectivorous bats are not important vectors of RABV in Argentina despite the great diversity of bat species and the extensive Argentinean territory.

**Methods:**

We studied the positivity rate of RABV detection in different areas of the country, and the antigenic and genetic diversity of 99 rabies virus (RABV) strains obtained from 14 species of insectivorous bats collected in Argentina between 1991 and 2008.

**Results:**

Based on the analysis of bats received for RABV analysis by the National Rabies system of surveillance, the positivity rate of RABV in insectivorous bats ranged from 3.1 to 5.4%, depending on the geographic location. The findings were distributed among an extensive area of the Argentinean territory. The 99 strains of insectivorous bat-related sequences were divided into six distinct lineages associated with *Tadarida brasiliensis*, *Myotis spp*, *Eptesicus spp*, *Histiotus montanus*, *Lasiurus blosseviilli* and *Lasiurus cinereus*. Comparison with RABV sequences obtained from insectivorous bats of the Americas revealed co-circulation of similar genetic variants in several countries. Finally, inter-species transmission, mostly related with *Lasiurus* species, was demonstrated in 11.8% of the samples.

**Conclusions:**

This study demonstrates the presence of several independent enzootics of rabies in insectivorous bats of Argentina. This information is relevant to identify potential areas at risk for human and animal infection.

## Author Summary

In Argentina, successful vaccination and control of terrestrial rabies in the 1980s revealed the importance of the aerial route in RABV transmission. Current distribution of cases shows a predominance of rabies by hematophagous bats in the Northern regions where rabies is a major public health concern; in contrast, in Central and Southern regions where rabies is not a major public health concern, little surveillance is performed. Based on the analysis of insectivorous bats received for RABV analysis by the National Rabies system of surveillance, the positivity rate of RABV in insectivorous bats in these regions ranged from 3.1 to 5.4%. This rate is comparable to other nations such as the United States (9–10%) where insectivorous bats are an important cause of concern for RABV surveillance systems. Antigenic and genetic analysis of a wide collection of rabies strains shows the presence of multiple endemic cycles associated with six bat insectivorous species distributed among an extensive area of the Argentinean territory and several countries of the Americas. Finally, inter-species transmission, mostly related with *Lasiurus* species, was demonstrated in 11.8% of the samples. Increased public education about the relationship between insectivorous bats and rabies are essential to avoid human cases and potential spread to terrestrial mammals.

## Introduction

Rabies is a fatal infection of the central nervous system primarily transmitted by rabid animal bites. Rabies virus (RABV) circulates through two different epidemiological cycles: terrestrial and aerial, where dogs, foxs or skunks and bats, respectively, act as most relevant reservoirs and/or vectors.

In Argentina, successful vaccination and control of canine rabies in the 1980s revealed the importance of bats in RABV transmission. Cases associated with the hematophagous vampire bat *Desmodus rotundus* are common in endemic areas of Argentina [1]. Two human rabies cases were associated with this species in 1997 and 2001 [2]. No cases have yet been associated with insectivorous bats, a stark contrast to the United States and Canada where these bats are the most common source of indigenously acquired human rabies infections [3]. Thus, it is widely accepted that insectivorous bats are not important vectors of RABV in Argentina despite the great diversity of bat species and the extensive Argentinean territory [4], [5], [6].

Monoclonal antibodies (N-Mabs) directed against the viral nucleoprotein (produced by the CDC, USA) have allowed for identification of antigenic variants (V) associated with insectivorous bats circulating in Argentina. Two variants were identified: V4 and V6, associated with *Tadarida brasiliensis* and *Lasiurus cinereus*, respectively [7], [8]. Additionally, although a limited number of specimens were analyzed, partial sequencing of the viral nucleoprotein has revealed at least four genetic variants or lineages associated with other insectivorous bat species [8]. Here we present an extensive study of the geographical distribution of disease associated with insectivorous bats, to infer which species are involved in the maintenance and transmission cycle of RABV in Argentina and to identify possible interspecies transmission patterns.

## Methods

### Passive surveillance of rabies from insectivorous bats

The Argentinean National Rabies Network consists of ten regional laboratories distributed in northern and central regions of the country. The south of Argentina is considered free of terrestrial rabies. RABV detection is mainly performed using direct immunofluorescence detection in brain samples and mouse inoculation test. Later, RABV isolates are sent to two National Reference Laboratories for antigenic characterization (DILACOT, SENASA or Instituto de Zoonosis “Dr Luis Pasteur”). Taxonomic characterization of the bats is performed by local specialists at the collection sites.

### Virus isolates and sequences

A total of 99 rabies samples were obtained from insectivorous bats and bat related cases between 1991 and 2008 from throughout Argentina. All viruses were isolated by intracerebral inoculation in mice as described previously [9]. Eight other RABV isolates from Argentinean insectivorous bats were also analyzed [8]. The host species and geographic location of rabies isolates are shown in **Table S1**. Samples were identified as follows: The first letters indicate abbreviation for the bat species follow by the number for each sample in the virus repository at SENASA or Instituto Pasteur and the year of collection.

In addition, four terrestrial specimens (Saldg126, Saldg146, Chadg120, Chafx119) and six vampire bat related cases (Ctebv01, Chabv90, Ctebv011, Ctehm82, Chabv72, Chabv129) were included in this study for comparison. In the comparator group, we included 93 sequences corresponding to historical samples available in GenBank, which represent the major bat RABV clades reported in the United States, Canada, Mexico and South America.

### Antigenic characterization

Antigenic characterization was performed by indirect immunofluorescence using a panel of eight monoclonal antibodies directed against the viral nucleoprotein (C1, C4, C9, C10, C12, C15, C18, C19) kindly provided by the Centers for Disease Control and Prevention, (Atlanta, GA, USA). Positivity reactivity results were analyzed using previously described antigenic variant patterns [7].

### RT-PCR and DNA sequencing

Viral RNA was extracted from isolates using TRIzol® (Invitrogen, Carlsbad, CA, USA). Reverse transcription and PCR amplification were achieved with primers 10 g and 304, as previously described [10]. The amplified product was sequenced using a BigDye Terminator v3.1 cycle sequencing kit according to the manufacturer's protocol with the ABI PRISM® 310 Genetic Analyzer (Applied Biosystems Inc. Foster City, California, USA).

### Phylogenetic analysis

A 264-bp region corresponding to the nucleoprotein gene located between nucleotides 1157 and 1420 and amino acids 363 to 450 (numbered according to strain SAD B19) was analyzed [11]. Raw sequence data were first edited using CHROMAS software (version1.3, Mc Carthy 1996, Griffith University, Queensland, Australia). Complete alignment was performed with Clustal X 1.8 [12]. The alignment was analyzed using Kimura 2 parameters as a method of substitution and Neighbor-Joining model to reconstruct the phylogenetic tree (MEGA version 4.1) [13]. The statistical significance of the phylogenies constructed was estimated by bootstrap analysis with 1000 pseudoreplicate data sets [14], [15].

### GenBank accession numbers

Partial nucleoprotein gene sequences described in this study were deposited in the GenBank database under the following accession numbers JF738250–JF738348.

## Results

### Geographic distribution of rabies in insectivorous bats

Between 1995 and 2007, public health authorities notified 1096 cases of animal rabies: dogs (33.6%), cattle (45.9%) or insectivorous bats (15.4%). The success of vaccination programs meant that of 200 rabies cases reported for 2008–09, canine rabies only represented 14.5%, while rabies transmitted by insectivorous bats increased to 38%, and cattle remained constant (45.5%) [16]. Geographic distribution of rabies in Argentina between 2008 and 2009 is shown in **Table 1**. Importantly, Northeastern (NE) and Northwestern (NW) regions show a predominance of rabies by vampires while in Central and South regions, almost all reported cases are associated with insectivorous bats.

**Table 1:** Regional distribution of rabies in Argentina, 2008–2009.

Species	Northeastern	Northwestern	Central	South	Total
Cattle	59	29	6	0	94
Insectivorous bats	2	3	70	4	79
Dog	4	23	0	0	27
Fox	3	0	0	0	3
Total	68	55	76	4	203

Argentina is divided into five epidemiological regions. Cuyo region has not reported cases of rabies in the period of this study.

From 1991 to 2008, Laboratories of National Rabies Network tested 4536 insectivorous bats for RABV from Buenos Aires city (CABA) and 23 from provinces of Argentina. Of these, 207 were found to be positive. Three provinces accounted for 82.2% of all reported cases of rabies in bats: Buenos Aires province, 70 cases (33.7%); CABA, 63 cases (30.4%); and Santa Fe province, 38 cases (18.4%). National positivity rate of rabies caused by insectivorous bats could not be calculated since the total number of bat specimens received from all provinces for rabies investigation was unavailable. However, local positivity rate could be determined in CABA, 3.5% (63/1792), Buenos Aires province 5.4% (70/1307) and Santa Fe province 3.1% (38/1113).

Of the bats infected with rabies virus, 65.4% of RABV-positive cases of *T. brasiliensis* were detected in CABA, while 77.8% of *L. cinereus* were detected in Buenos Aires province, and 60.0% of *Myotis* genus and 87.5% of *Eptesicus* genus were found by Santa Fe province. Great diversity was observed in Santa Fe (ten species) and Buenos Aires (nine species). *T. brasiliensis* accounted for 92.7% of the total in CABA (four species).

### Antigenic typing

Antigenic typing was performed on 103 isolates (**Table S1**). Antigenic variant 4 (AgV4) was identified in 57 bats: 53 (92.9%) in *T.brasiliensis*; the rest (7.1%) in *M.molossus (n = 2)*, *Eumops patagonicus (n = 1)* and unidentified bat (n = 1). This variant showed the greatest distribution, scattered throughout the provinces. Antigenic variant 6 (AgV6) was identified in 25 specimens: 11 *L.cinereus* (44.0%), 4 *L.ega* (16.0%), and the remaining 10 (40.0%) in *Eptesicus spp* (n = 1), *M.molossus* (n = 1), *M.levis* (n = 1), *M.nigricans (n = 1)*, one *Myotis spp* (n = 1), four unidentified bats, and one dog. This variant mainly circulated in central provinces. Finally, 22 isolates exhibited 11 different atypical reaction pattern (ARP) (**Table 2**). These isolates were from *Myotis* (n = 7), *Eptesicus* (n = 9), *Histiotus* genus (n = 3), *T. brasiliensis* (n = 1), *L. blossevillii* (n = 1) bats and one from a cat.

**Table 2:** Antigenic patterns of bat rabies viruses from Argentina.

Associated source or reservoir^a^	Patterns of reaction (N-Mabs)								Antigenic Variant
	C1	C4	C9	C10	C12	C15	C18	C19	
Dog/mongoose	+	+	+	+	+	+	−	+	V1
Dog	+	+	−	+	+	+	−	+	V2
*Desmodus rotundus*	−	+	+	+	+	−	−	+	V3
*Tadarida brasiliensis*	−	+	+	+	+	−	−	−	V4
*Lasiurus cinereus*	+/−	+	+	+	+	−	−	−	V6
LB	+/−	+	+	+	+/−	−	−	−	ARP^b^
MY1	+	+	+	+	+	−	−	−	ARP
MY2	−	+	−	+	+	−	−	+	ARP
MY3	−	+	+	+	+	+	+	+	ARP
MY4	−	+	+	+	−	−	−	−	ARP
MY5	−	+	+/−	+	+	+	−	−	ARP
HM1	−	+	−	+	−	−	−	−	ARP
HM2	−	+	−	+	+	−	−	−	ARP
EP1	+	+	−	+	+	−	−	−	ARP
EP2	−	+/−	−	+/−	+	−	−	−	ARP
EP3	−	+	−	+	+	−	−	−	ARP

a LB, *Lasiurus blosseviilli* reactivity pattern found for sample Lb658-BA03; MY1–5, *Myotis* spp. reactivity pattern found for samples Epf457-SF04 and My594-CHA05 (MY1), Bamsbt121 (MY2), Myl15M-CBA03 (MY3), Sfemnbt116 (MY4) and Myn140-SF97 (MY5); HM1–2, *Histiotus montanus* reactivity pattern found for samples Stchmbt80 (HM1) and Hm620-CHU07 and Hm580CHU07 (HM2); EP1–3, *Eptesicus* spp reactivity pattern found for samples Epf1288-SF08 and Epf787-SF07 (EP1), Epf1202-SF06 and Epb497-SF05 (EP2) and Epf062-BA03 and Epb458-SF05 (EP3).

b ARP, atypical reaction pattern.

### Molecular characterization

**Figure 1:**
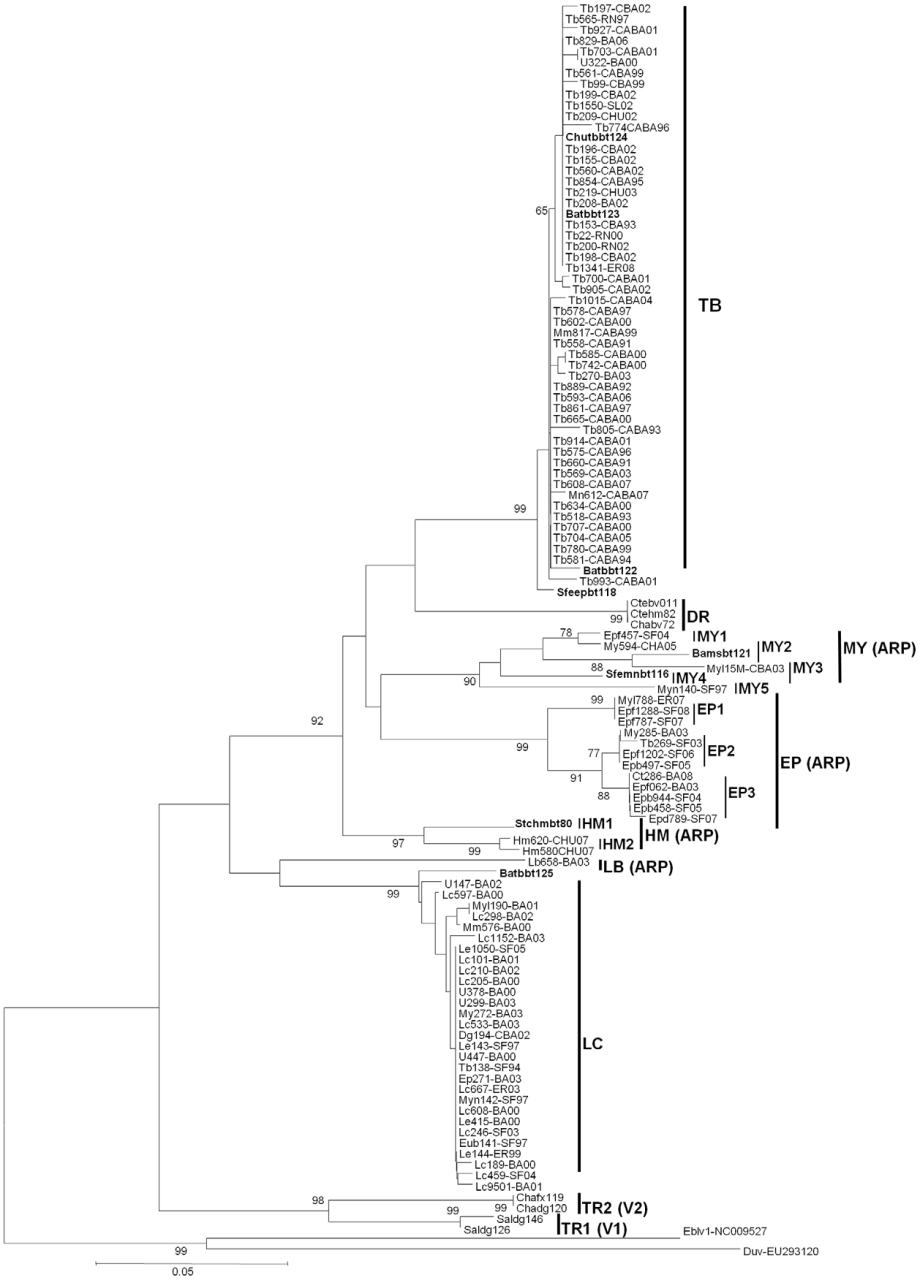
Phylogenetic relationships among Argentinean isolates. Analysis was based on 264 nt of the nucleoprotein gene. A tree was constructed based on Kimura and neighbor joining parameters. TB, *Tadarida brasiliensis*; DR, *Desmodus rotundus*; MY, *Myotis spp.*; HM, *Histiotus montanus*; EP, *Eptesicus spp*; LB, *Lasiurus blosseviilli*; LA, *Lasiurus cinereus*; TR, Terrestrial.

A total of 107 insectivorous bat-related sequences segregated into six distinct lineages. This was well supported by significant bootstrap values and clearly differentiated from those related to rabies in terrestrial animals (**Figure 1**).

**Figure 2:**
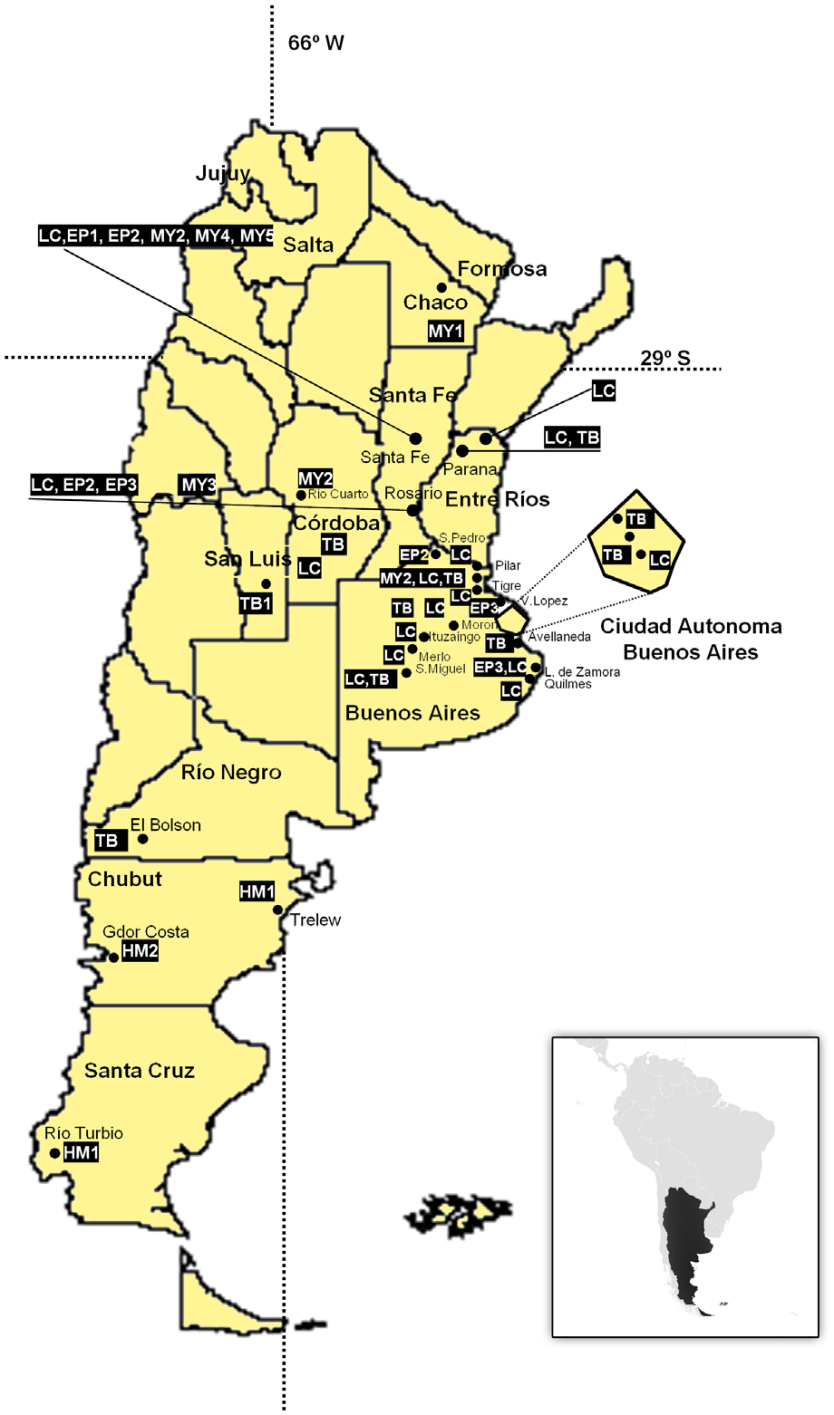
Geographic distribution of RABV sublineages associated with insectivorous bats from Argentina.

The first lineage (TB) included 51 samples from *T. brasiliensis*, two from *M.molossus*, one from *Eumops patagonicus* and one from an unidentified bat. All isolates were typed as V4. This lineage exhibited a high nucleotide and amino acid similarity (99.0% and 100.0%, respectively). It showed the amino acidic residue N_394_ in the nucleoprotein that is characteristic of this genetic variant (**Table 3**). Rabies isolates obtained from *Myotis*, *Eptesicus* and *Histiotus* bats grouped in three highly diverse lineages (nucleotide intra-lineage distance of 6.7%, 2.0% and 3.4%, respectively). All these isolates resulted in several atypical reaction patterns with N-Mabs (ARP). *Myotis* lineage (MY) was subsequently divided in five sublineages (MY1–5) less-well supported, scattered throughout central provinces (**Figure 2**). The *Eptesicus* lineage (EP) was also divided in three sublineages (named EP1–3). The three EP sublineages resulted in different ARP circulating in Santa Fe, Entre Rios and Buenos Aires. Both lineages MY and EP presented a similar coding signature containing the amino acids A_385_ and L_419_. Lastly, the *Histiotus montanus* lineage (HM) included three isolates from southern provinces (Santa Cruz and Chubut). It was further divided in two sublineages (HM1 and HM2) in correspondence with their antigenic and nucleotide sequence clustering with nucleotide and amino acid distance of 4.3% and 2.3%; amino acid difference at position 369 (Q_369_K). Furthermore, they showed different reaction with C12 N-Mab (**Table 3**).

**Table 3:** Consensus amino acid sequences of bat rabies viruses from Argentina.

GeneticVariant	Amino acid sequence																		
	369	371	374	377	378	379	385	390	394	397	407	410	414	418	419	433	436	443	450
**Consensus**	**Q**	**A**	**L**	**T**	**E**	**V**	**G**	**D**	**Y**	**S**	**T**	**M**	**G**	**R**	**G**	**A**	**N**	**N**	**S**
TR1					D					G									
TR2	R				D					G							G		
DR				A		T													
TB						M			N										
LC													S						
LB													S		T				
MY1					D		A							K	L				
MY2			S		D	E	A	E					V		L				
MY3			S		D	A	A	E			N	I		K	L				
MY4			S		D		A								L				
MY5							A								L				
HM1																			
HM2	K																		P
EP1	K					A	A	E							L	S			
EP2	K					A	A								L	S		S	
EP3	K					A	A								L	S		S	

The last 88 amino acids encoded by the rabies virus nucleoprotein gene at the carboxy terminus are shown.

Finally, the last two lineages (named LB and LC), were detected in three different species of *Lasiurus* bats. LB included a single sample of *L.blossevilli*, antigenically typed as ARP. LC included 30 samples, obtained from 13 *L.cinereus* (43.3%), four *L.ega* (13.3%) and others species: one *Eptesicus spp.*, one *E.bonariensis*, one *M.molossus*, three *Myotis spp.*, one dog, and four samples from unclassified bats. LC is one of the less divergent lineages (0.3%) and was formed from 30 samples. Virus of the lineage LC were mainly typed as V6 (n = 25, 83,3%); ARP (n = 4, 13,3%) and V4 (n = 1; 3,4%) Deduced amino acid sequences revealed a characteristic amino acid S_414_ in both LC and LB lineages. Moreover, a change at position 419 (S_419_T) was identified between LC and LB lineages.

### Molecular relationships between rabies associated with insectivorous bats of Argentina and the Americas

**Figure 3:**
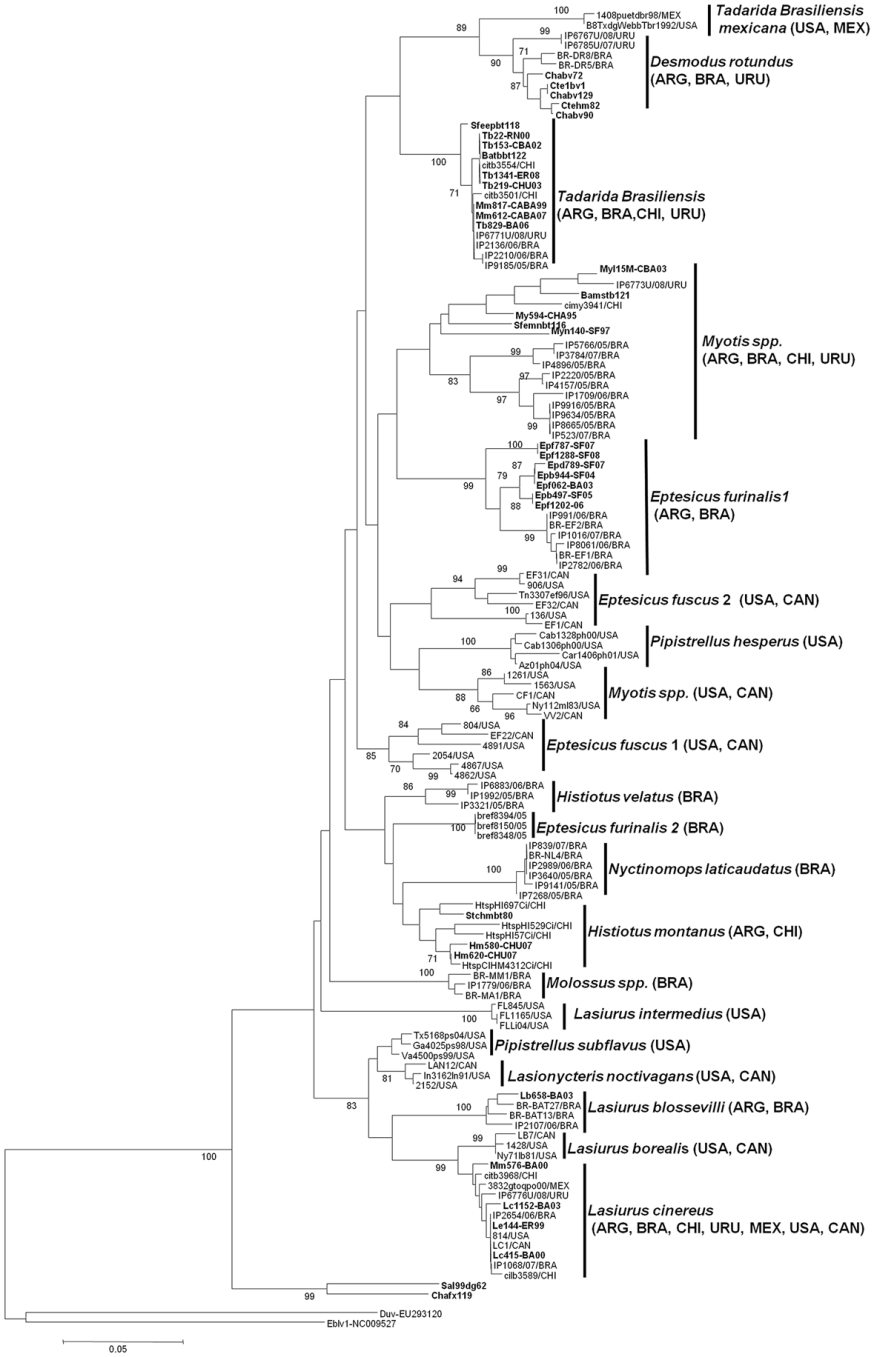
Phylogenetic tree of insectivorous bats rabies from Americas. Tree was generated of N-J method (Kimura two-parameter model) of a portion of the N gene coding sequence (264 nt). Bold font indicates RABV samples from Argentinean bats analyzed in this study. The geographical origins of comparator group sequences are included in the name of each sample. Sample numbers appeared as GenBank database.

Analysis of nucleoprotein RABV sequences associated with insectivorous bats in Argentina and the Americas revealed several monophyletic clusters associated with specific bat species. This was consistent with previous analyses [17], [18], [19] (**Figure 3**). RABV samples obtained from *T. brasiliensis* bats from Argentina, Brazil, Chile and Uruguay segregated into a monophyletic cluster. RABV sequences associated with *Myotis* species occurring in South America were divided in two heterogeneous lineages formed by samples from Argentina, Chile and Uruguay or Brazil. Instead, RABV sequences associated with *Myotis* species occurring in North America (USA, Canada) formed a different cluster. Similarly, RABV samples from *Eptesicus* bats of Argentina and Brazil, represented primarily by *furinalis* subspecies, were grouped in two clusters. These clusters did not show relationships with the genetic clusters associated with *fuscus* subspecies from USA and Canada. Strains that were recovered in Argentina and Chile from *Histiotus* bats circulated in both countries as at least two sublineages less-well supported. Finally, RABV sequences obtained from different species of *Lasiurus* from the Americas were divided in three monophyletic clusters associated with subspecies *blossevillii*, *borealis* and *cinereus/ega*. An Argentinean sample obtained from *L. blossevillii* (Lb658-BA03) grouped with two Brazilian *L. blossevillii*-related RABV strains (BR-BAT27 and BR-BAT13). RABV isolates obtained from *L. cinereus* and *L. ega* from Argentina, Brazil, Chile, Uruguay, USA, Mexico and Canada grouped in a cluster with the highest nucleotide homogeneity (99.5%).

## Discussion

The current understanding of the epidemiology of rabies in Argentina states that the vast majority of cases in the Northern region are associated with hematophagous bats. Very few cases of terrestrial rabies were also detected in the Northwestern (Salta and Jujuy) and Northeastern provinces (Formosa and Chaco) during 2010. The rest of the country has been considered terrestrial rabies-free since the early 1980s. This epidemiological situation makes it difficult to estimate the real impact of the insectivorous bat rabies in our country. In the north, very few cases have been detected probably masked by endemic bovine rabies. While in the center and south, the absence of terrestrial rabies has led to low level of awareness among general public, public health officials and health administrators. As a result, rabies associated with insectivorous bats and its potential consequent implications in public and animal health have been largely neglected in Argentina.

Our study reveals that the positivity rate of rabies in insectivorous bats received in the laboratory for analysis ranges from 3.1 to 5.4%. This proportion is comparable to other countries such as the United States (9–10%) where insectivorous bats are the only cause of concern for RABV surveillance systems, and other South American countries (Brazil (1.3%) and Chile (4.2%) [20], [21], [22]. Fortunately, <1% of natural bat population have been shown to be infected [23]. Thus, the risk of contracting rabies from insectivorous bats is low. However, evidence indicates that many of the human cases of rabies resulted from exposures to bats that were not recognized or reported [3]. Consequently, prevention of human infection with bat rabies virus variants remains an important public health concern. On the other hand, emergence of rabies in terrestrial hosts after spillover from chiropteran reservoirs has been described but does not typically result in sustained transmission. However, if host switching of rabies virus variants occur, once established could be become enzootic in new reservoir species [24]. Therefore, special attention should be paid to unusual epidemiological patterns of terrestrial rabies transmission in new geographic areas.

Antigenic characterization utilizing the eight monoclonal antibodies developed by the CDC is widely used in Latin America for RABV surveillance. However, in some instances, antigenic analysis is unable to identify RABV isolates obtained from several insectivorous bat species because these isolates produced atypical reaction patterns (unrelated to previously described virus reservoirs) [8]. In those cases, partial genetic analysis of the viral nucleoprotein sequence allowed further characterization [25] allowing the identification of lineages or genetic variants maintained by insectivorous bat species in an independent enzootic cycle. Indeed, in our work, we identified six RABV lineages that were specifically associated with specific bat species. Moreover, genetic analysis allowed us to differentiate some of the previously accepted antigenic variants in independent sublineages that appear to be related with different geographical or ecological niche behaviors. *Tadarida brasiliensis* maintains circulation of its own antigenic (AgV4) with high degree of nucleotide and amino acid homogeneity in Argentina, Chile, Brazil and Uruguay. In contrast, analysis of RABV isolates recovered from *Myotis* and *Eptesicus* species showed a high antigenic diversity that could be related to the gregarious and non-migratory habits of these species [17], [18]. The elevated antigenic diversity of RABV sustained by *Eptesicus* or *Myotis* species can complicate definitive strain typing. This could be the case of the only Myotis associated strain from Chile, which was assigned to an apparent AgV3 using a reduced panel of eight N-MAbs by Yung et al. [26], but our genetic characterization revealed its real clustering into Myotis group.

All Argentinean isolates obtained from *Histiotus montanus*, clustered in a single genetic group along with strains from Chile confirming that this species is its own the viral variant reservoir. There is little information about this species other than it lives a solitary life and migrates seasonally between Argentina and Chile [27]. Although both countries are separated by the Andes Chain, an important natural barrier, low-lying passages of the mountain allow different species of bats and terrestrial mammals to move between the countries and thus spreading this viral variant [28].

Members of the genus *Lasiurus* typically are solitary and migratory bats. In our study, rabies samples from *L. blossevillii*, *cinereus* and *ega* were analyzed. A RABV isolate from Argentinean *L.blossevillii* bat was distant to others obtained from the Lasiurus genus but clustered with others from the same bat species from Brazil. Indeed, it yields a previously unrecognized genetic lineage circulating in Argentina.

On the other hand, antigenic and molecular analysis of rabies isolates from *Lasiurus cinereus* confirmed that this species maintained its own antigenic (V6) and genetic variant as previously reported [29]. Despite showing a wide geographical distribution (Canada to Argentina), RABV isolates from this species exhibited a high degree of nucleotide and amino acid homogeneity, which could be explained by its ability to transport its own specific strain during its long migration pattern. Two rabies samples of Chilean Lasiurus have been assigned to AgV4 (*T.brasiliensis* reservoir) by Yung et al. [26], but our phylogenetic analysis showed that they grouped with Lasiurus strains (AgV6). This apparent discrepancy could be explained by the inadequate use of the N-Mabs panel which could lead to confusion between AgVs. The difference between both variants falls only in the reactivity with monoclonal C1. According to the N-Mabs manufacturer's instructions, all negative or diminished reactions should be confirmed by furthers tests of the samples with a 10-fold less-dilute antibody [30].

Molecular characterization of RABV isolates revealed that inter-species transmission is a relatively common event. Eleven (11.8%) of the 93 bat samples tested showed to be infected by a variant supported by another bat species. Cross-species transmission is facilitated by several types of species life-history traits and perhaps environmental variables structuring communities [31]. In the case of *Lasiurus*, these seem to have an important role in these events, since we have identified this variant nearly in all bat species studied. Although, *L.cinereus* generally roosts in isolation, it has been observed occasional aggressive encounters at share roosts or during flight, which could promote viral transmission.

The findings of this study demonstrate the presence of rabies in several species of insectivorous bats throughout Argentina. Phylogenetic analysis of an extensive collection of rabies strains obtained from 14 species over a 17-year period shows complex epidemiological patterns characterized by the presence of multiple endemic cycles and relatively frequent inter-species transmission that are affected by several ecological aspects such as migration patterns, roosting and habitat. The establishment of viral variants associated with specific bat species can assist in the epidemiological investigation of cases of human rabies associated with bats and potential events spread to terrestrial mammals.

## Supporting Information

Table S1 **Rabies isolates from insectivorous bats of Argentina.** ARP: atypical reaction pattern; ND: not done.
(DOC)
