# Supplementary material for: High Diversity of Rabies Viruses Associated with Insectivorous Bats in Argentina: Presence of Several Independent Enzootics
Source: PLoS Negl Trop Dis. 2012 May 8;6(5):e1635. doi: 10.1371/journal.pntd.0001635 (PMC3348165; doi:10.1371/journal.pntd.0001635)
Supplement: Table S1 — Rabies isolates from insectivorous bats of Argentina. ARP: atypical reaction pattern; ND: not done. (DOC) [file pntd.0001635.s001.doc]

**Table 1. Rabies isolates from insectivorous bats of Argentina**

| **Isolates** | **City, Province** | **Species** | **Date** | **Antigenic variant** | **Genetic Variant** | **GenBank**  **Accession no.** |
| --- | --- | --- | --- | --- | --- | --- |
| Tb558-CABA91 | Buenos Aires City | *Tadarida brasiliensis* | 1991 | 4 | TB | JF738266 |
| Tb660-CABA91 | Buenos Aires City | *Tadarida brasiliensis* | 1991 | 4 | TB | JF738259 |
| Tb889-CABA92 | Buenos Aires City | *Tadarida brasiliensis* | 1992 | 4 | TB | JF738261 |
| Tb518-CABA93 | Buenos Aires City | *Tadarida brasiliensis* | 1993 | 4 | TB | JF738265 |
| Tb805-CABA93 | Buenos Aires City | *Tadarida brasiliensis* | 1993 | 4 | TB | JF738268 |
| Tb581-CABA94 | Buenos Aires City | *Tadarida brasiliensis* | 1994 | 4 | TB | JF738257 |
| Tb854-CABA95 | Buenos Aires City | *Tadarida brasiliensis* | 1995 | 4 | TB | JF738279 |
| Tb774-CABA96 | Buenos Aires City | *Tadarida brasiliensis* | 1996 | 4 | TB | JF738291 |
| Tb575-CABA96 | Buenos Aires City | *Tadarida brasiliensis* | 1996 | 4 | TB | JF738253 |
| Tb578-CABA97 | Buenos Aires City | *Tadarida brasiliensis* | 1997 | 4 | TB | JF738264 |
| Tb861-CABA97 | Buenos Aires City | *Tadarida brasiliensis* | 1997 | 4 | TB | JF738255 |
| Tb565-RN97 | El Bolson, Rio Negro | *Tadarida brasiliensis* | 1997 | 4 | TB | JF738281 |
| Mn817-CABA99 | Buenos Aires City | *Molossus molossus* | 1999 | 4 | TB | JF738258 |
| Tb561-CABA99 | Buenos Aires City | *Tadarida brasiliensis* | 1999 | 4 | TB | JF738286 |
| Tb780-CABA99 | Buenos Aires City | *Tadarida brasiliensis* | 1999 | 4 | TB | JF738260 |
| Tb99-CBA99 | Cordoba, Cordoba | *Tadarida brasiliensis* | 1999 | 4 | TB | JF738290 |
| U322-BA00 | San Miguel, Buenos Aires | Unclassified | 2000 | 4 | TB | JF738272 |
| Tb585-CABA00 | Buenos Aires City | *Tadarida brasiliensis* | 2000 | 4 | TB | JF738269 |
| Tb602-CABA00 | Buenos Aires City | *Tadarida brasiliensis* | 2000 | 4 | TB | JF738256 |
| Tb634-CABA00 | Buenos Aires City | *Tadarida brasiliensis* | 2000 | 4 | TB | JF738263 |
| Tb665-CABA00 | Buenos Aires City | *Tadarida brasiliensis* | 2000 | 4 | TB | JF738252 |
| Tb707-CABA00 | Buenos Aires City | *Tadarida brasiliensis* | 2000 | 4 | TB | JF738254 |
| Tb742-CABA00 | Buenos Aires City | *Tadarida brasiliensis* | 2000 | 4 | TB | JF738270 |
| TB2-RN00 | El Bolson, Rio Negro | *Tadarida brasiliensis* | 2000 | 4 | TB | JF738283 |
| Tb700-CABA01 | Buenos Aires City | *Tadarida brasiliensis* | 2001 | 4 | TB | JF738273 |
| Tb703-CABA01 | Buenos Aires City | *Tadarida brasiliensis* | 2001 | 4 | TB | JF738289 |
| Tb914-CABA01 | Buenos Aires City | *Tadarida brasiliensis* | 2001 | 4 | TB | JF738267 |
| **Isolates** | **City, Province** | **Species** | **Date** | **Antigenic variant** | **Genetic Variant** | **GenBank**  **Accession no.** |
| Tb927-CABA01 | Buenos Aires City | *Tadarida brasiliensis* | 2001 | 4 | TB | JF738276 |
| Tb993-CABA01 | Buenos Aires City | *Tadarida brasiliensis* | 2001 | 4 | TB | JF738333 |
| Batbbt122 | La Matanza, Buenos Aires | *Tadarida brasiliensis* | 2001 | 4 | TB |  |
| Batbbt123 | 3 de febrero, Buenos Aires | *Tadarida brasiliensis* | 2001 | 4 | TB |  |
| Batbbt125 | 3 de febrero, Buenos Aires | *Tadarida brasiliensis* | 2001 | 4 | LC |  |
| Sfeepbt118 | Rosario, Santa Fe | *Eumops patagonicus* | 2001 | 4 | EU |  |
| Chutbbt124 | Puerto Madryn, Chubut | *Tadarida brasiliensis* | 2001 | 4 | TB |  |
| TB08-BA02 | Moron, Buenos Aires | *Tadarida brasiliensis* | 2002 | 4 | TB | JF738282 |
| Tb560-CABA02 | Buenos Aires City | *Tadarida brasiliensis* | 2002 | 4 | TB | JF738278 |
| Tb905-CABA02 | Buenos Aires City | *Tadarida brasiliensis* | 2002 | 4 | TB | JF738274 |
| TB09-CHU02 | Puerto Madryn, Chubut | *Tadarida brasiliensis* | 2002 | 4 | TB | JF738340 |
| TB96-CBA02 | Cordoba, Cordoba | *Tadarida brasiliensis* | 2002 | 4 | TB | JF738277 |
| TB97-CBA02 | Cordoba, Cordoba | *Tadarida brasiliensis* | 2002 | 4 | TB | JF738275 |
| TB98-CBA02 | Cordoba, Cordoba | *Tadarida brasiliensis* | 2002 | 4 | TB | JF738287 |
| TB99-CBA02 | Cordoba, Cordoba | *Tadarida brasiliensis* | 2002 | 4 | TB | JF738288 |
| TB53-CBA02 | Cordoba, Cordoba | *Tadarida brasiliensis* | 2002 | 4 | TB | JF738284 |
| TB55-CBA02 | Cordoba, Cordoba | *Tadarida brasiliensis* | 2002 | 4 | TB | JF738285 |
| TB00-RN02 | El Bolson, Rio Negro | *Tadarida brasiliensis* | 2002 | 4 | TB | JF738342 |
| TB550-SL02 | Villa Mercedes, San Luis | *Tadarida brasiliensis* | 2002 | 4 | TB | JF738280 |
| TB70-BA03 | Tigre, Buenos Aires | *Tadarida brasiliensis* | 2003 | 4 | TB | JF738271 |
| Tb569-CABA03 | Buenos Aires City | *Tadarida brasiliensis* | 2003 | 4 | TB | JF738262 |
| TB19-CHU03 | Puerto Madryn, Chubut | *Tadarida brasiliensis* | 2003 | 4 | TB | JF738341 |
| TB015-CABA04 | Buenos Aires City | *Tadarida brasiliensis* | 2004 | 4 | TB | JF738343 |
| Tb704-CABA05 | Buenos Aires City | *Tadarida brasiliensis* | 2005 | 4 | TB | JF738344 |
| Tb829-BA06 | Avellaneda, Buenos Aires | *Tadarida brasiliensis* | 2006 | 4 | TB | JF738336 |
| Tb593-CABA06 | Buenos Aires City | *Tadarida brasiliensis* | 2006 | 4 | TB | JF738338 |
| Tb608-CABA07 | Buenos Aires City | *Tadarida brasiliensis* | 2007 | 4 | TB | JF738345 |
| Mm612-CABA07 | Buenos Aires City | *Molossus molossus* | 2007 | 4 | TB | JF738346 |
| TB341-ER08 | Parana, Entre Rios | *Tadarida brasiliensis* | 2008 | 4 | TB | JF738339 |
| **Isolates** | **City, Province** | **Species** | **Date** | **Antigenic variant** | **Genetic Variant** | **GenBank**  **Accession no.** |
| Le143-SF97 | Santa Fe, Santa Fe | *Lasiurus ega* | 1997 | 6 | LA | JF738319 |
| MyN142-SF97 | Rosario, Santa Fe | *Myotis nigricans* | 1997 | 6 | LA | JF738321 |
| Le144-ER99 | Parana, Entre Rios | *Lasiurus ega* | 1999 | 6 | LA | JF738320 |
| Lc597-BA00 | Villa Ballester, Buenos Aires | *Lasiurus cinereus* | 2000 | 6 | LA | JF738330 |
| Lc608-BA00 | La Lucila, Buenos Aires | *Lasiurus cinereus* | 2000 | 6 | LA | JF738309 |
| Lc189-BA00 | Ituzaingo, Buenos Aires | *Lasiurus cinereus* | 2000 | 6 | LA | JF738313 |
| Lc205-BA00 | Ituzaingo, Buenos Aires | *Lasiurus cinereus* | 2000 | 6 | LA | JF738325 |
| Le415-BA00 | Moron, Buenos Aires | *Lasiurus ega* | 2000 | 6 | LA | JF738311 |
| Mn576-BA00 | Moron, Buenos Aires | *Molossus molossus* | 2000 | 6 | LA | JF738329 |
| U378-BA00 | Merlo, Buenos Aires | Unclassified | 2000 | 6 | LA | JF738310 |
| U447-BA00 | Lomas de Zamora, Buenos Aires | Unclassified | 2000 | 6 | LA | JF738308 |
| Lc9501-BA01 | Moron, Buenos Aires | *Lasiurus cinereus* | 2001 | 6 | LA | JF738324 |
| Lc101-BA01 | San Miguel, Buenos Aires | *Lasiurus cinereus* | 2001 | 6 | LA | JF738312 |
| Myl190-BA01 | Tres de Febrero, Buenos Aires | *Myotis levis* | 2001 | 6 | LA | JF738327 |
| Lc210-BA02 | San Isidro, Buenos Aires | *Lasiurus cinereus* | 2002 | 6 | LA | JF738318 |
| Lc533-BA02 | San Miguel, Buenos Aires | *Lasiurus cinereus* | 2002 | 6 | LA | JF738323 |
| Lc298-BA02 | Pilar, Buenos Aires | *Lasiurus cinereus* | 2002 | 6 | LA | JF738328 |
| U147-BA02 | Pilar, Buenos Aires | Unclassified | 2002 | 6 | LA | JF738331 |
| Dg194-CBA02 | Cordoba, Cordoba | Canine | 2002 | 6 | LA | JF738304 |
| Ep271-BA03 | Quilmes, Buenos Aires | *Eptesicus spp* | 2003 | 6 | LA | JF738307 |
| Lc1152-BA03 | San Miguel, Buenos Aires | *Lasiurus cinereus* | 2003 | 6 | LA | JF738326 |
| My272-BA03 | Vicente Lopez, Buenos Aires | *Myotis spp* | 2003 | 6 | LA | JF738315 |
| U299-BA03 | Tigre, Buenos Aires | Unclassified | 2003 | 6 | LA | JF738314 |
| Lc667-ER03 | Victoria, Entre Rios | *Lasiurus cinereus* | 2003 | 6 | LA | JF738306 |
| Le1050-SF05 | Santa Fe, Santa Fe | *Lasiurus ega* | 2005 | 6 | LA | JF738305 |
| Myn140-SF97 | Santo Tome, Santa Fe | *Myotis nigricans* | 1997 | ARP | MY5 | JF738303 |
| Stchmbt80 | Rio Turbio, Santa Cruz | *Histiotus montanus* | 2000 | ARP | HM1 |  |
| Bamsbt121 | 3 de febrero, Buenos Aires | *Myotis* spp. | 2001 | ARP | MY2 |  |
| **Isolates** | **City, Province** | **Species** | **Date** | **Antigenic variant** | **Genetic Variant** | **GenBank**  **Accession no.** |
| Sfemnbt116 | Rosario, Santa Fe | *Myotis nigricans* | 2001 | ARP | MY4 |  |
| Epf062-BA03 | Vicente Lopez | *Eptesicus furinalis* | 2003 | ARP | EP3 | JF738348 |
| My285-BA03 | San Pedro, Buenos Aires | *Myotis spp* | 2003 | ARP | EP2 | JF738297 |
| Lb658-BA03 | Buenos Aires City | *Lasiurus blossevillii* | 2003 | ARP | LB | JF738332 |
| Myl15M-CBA03 | Rio Cuarto, Cordoba | *Myotis levis* | 2003 | ARP | MY2 | JF738302 |
| TB69-SF03 | Santa Fe, Santa Fe | *Tadarida brasiliensis* | 2003 | ARP | EP2 | JF738295 |
| Epb944-SF04 | Rosario, Santa Fe | *Eptesicus brasiliensis* | 2004 | ARP | EP2 | JF738293 |
| Epf457-SF04 | Santa Fe, Santa Fe | *Eptesicus furinalis* | 2004 | ARP | MY1 | JF738300 |
| My594-CHA05 | Resistencia, Chaco | *Myotis spp* | 2005 | ARP | MY1 | JF738301 |
| Epb497-SF05 | Santo Tome, Santa Fe | *Eptesicus brasiliensis* | 2005 | ARP | EP2 | JF738296 |
| Epb458-SF05 | Rosario, Santa Fe | *Eptesicus brasiliensis* | 2005 | ARP | EP3 | JF738292 |
| Epf1202-06 | Santa Fe, Santa Fe | *Eptesicus furinalis* | 2006 | ARP | EP2 | JF738294 |
| Hm580-CHU07 | Gobernador Costa, Chubut | *Histiotus montanus* | 2007 | ARP | HM2 | JF738251 |
| Hm620-CHU07 | Trelew, Chubut | *Histiotus montanus* | 2007 | ARP | HM2 | JF738250 |
| Myl788-ER07 | Parana, Entre Rios | *Myotis levis* | 2007 | ARP | EP1 | JF738299 |
| Epd789-SF07 | Rosario, Santa Fe | *Eptesicus diminutus* | 2007 | ARP | EP3 | JF738335 |
| Epf787-SF07 | Santa Fe, Santa Fe | *Eptesicus furinalis* | 2007 | ARP | EP1 | JF738298 |
| Ct286-BA08 | Lomas de Zamora, Buenos Aires | Feline | 2008 | ARP | EP3 | JF738337 |
| Epf1288-SF08 | Santa Fe, Santa Fe | *Eptesicus furinalis* | 2008 | ARP | EP1 | JF738347 |
| TB38-SF94 | Rosario, Santa Fe | *Tadarida brasiliensis* | 1994 | ND | LA | JF738322 |
| Eub141-SF97 | Santa Fe, Santa Fe | *Eumops bonariensis* | 1997 | ND | LA | JF738316 |
| Lc246-SF03 | Santa Fe, Santa Fe | *Lasiurus cinereus* | 2003 | ND | LA | JF738317 |
| Lc459-SF04 | Rosario, Santa Fe | *Lasiurus cinereus* | 2004 | ND | LA | JF738334 |
